# The Yearly Prevalence of Findings in Endoscopy of the Lower Part of the Gastrointestinal Tract

**DOI:** 10.5402/2012/527634

**Published:** 2012-12-27

**Authors:** R. J. L. F. Loffeld, B. Liberov, P. E. P. Dekkers

**Affiliations:** Department of Internal Medicine and Gastroenterology, Zaans Medisch Centrum, P.O. Box 210, 1500 EE Zaandam, The Netherlands

## Abstract

*Introduction*. Endoscopy of the colon and rectum is increasingly used. *Aim of the Study*. All consecutive endoscopies of the colon and rectum were studied in order to assess the yearly prevalence of significant endoscopic diagnoses. *Methods*. All consecutive endoscopies of the colon and rectum were included. Endoscopies were done with endoscopes of Olympus. Significant endoscopic diagnoses were defined as colorectal cancer, polyps, diverticuli, large sessile polyps, and inflammatory bowel disease. *Results*. In 20 years a total of 24431 endoscopies were done. The yearly number of sigmoidoscopies was mean 96, range of 42–370. The number of colonoscopies was mean 1126, range of 643–1912. The number of colonoscopies significantly increased. The number of colonoscopies on request of an internist or gastroenterologist showed a slow but steady increase. Successful caecal intubation rose from 70% to 92% in 2011. Since 1996 there is a steep increase in the percentage of procedures with abnormalities. The number of cancer and polyps increased in twenty years. No great changes were seen in inflammatory bowel disease. *Conclusion*. Colonoscopy is a procedure with a high diagnostic yield. The number of patients with tumours rose in twenty years.

## 1. Introduction

Endoscopy of the colon and rectum is increasingly used as a diagnostic tool as well as therapeutic modality in diagnosis and treatment of abnormalities in the lower part of the gastrointestinal tract. Many studies have been published on inflammatory bowel disease, colorectal cancer, adenomas, and screening. Hence, prevalence and incidence of specific diseases are well known. Also results of screening colonoscopy in more or less asymptomatic individuals are known. Most studies include specific subsets of patients in a specific time period. 

Sparse data is known on the prevalence of findings of endoscopy of the colon and rectum in normal daily practice in nonselected patients. It is not known whether the yield of colonoscopy and sigmoidoscopy and hence morbidity patterns in patients show any change in consecutive years.

For this reason all consecutive endoscopies of the colon and rectum done in a single centre were studied in order to assess the yearly prevalence of significant endoscopic diagnoses. The present study is an extension of a previous paper [1]. 

## 2. Methods

All consecutive endoscopies of the lower gastrointestinal tract done at the Endoscopy Department of the Zaans Medical Centre, the regional hospital of the Zaanstreek region in The Netherlands, were included. 

From 1992 to 2006 three endoscopists (two gastroenterologists and one internist) did all endoscopies in nine weekly shifts. In September 2006 the staff was expanded with one gastroenterologist. Due to retirement of two endoscopists in 2008, two gastroenterologists did the procedures in 2008 and 2009. Since April 2010 a third gastroenterologist started clinical practice. 

In November 2009 an open-access facility for colonoscopy was started. General practitioners can request a colonoscopy on specified clinical indications without prior consultation by a gastroenterologist or internist. 

All endoscopies were done with endoscopes of Olympus (Olympus Zoetermeer, The Netherlands). In the beginning of the nineties nineties fibre-optic endoscopes were used. In 1993 video-endoscopes (EVIS 100 system) was introduced, and from 2000 the procedures were done with the EXERA 120 and later on 180 system.

All endoscopy reports were noted in a written report and subsequently prospectively put in a computerised system. Since the beginning of 2003 a customised computerised data system based on Endobase from Olympus was used.

Patients underwent endoscopy after a standard cleansing protocol. In the nineties cleansing was done with a PEG solution applied via a nasogastric tube or via Dulcolax, Prunacolon, and an enema prior to endoscopy. Scince 1995 ambulatory cleansing with four litres of Klean-prep and scince 2005 with two litres of Moviprep was used.

In the present paper only data on significant endoscopic diagnoses, defined as colorectal cancer, polyps, diverticuli, large sessile polyps, and inflammatory bowel disease, are reported. The remainder like lipoma, colon varices, solitary rectal ulcer syndrome, haemorrhoids, and so forth was defined as nonsignificant for the sake of the study and is not explicitly noted in the present study. 

Trend lines in the figures were drawn with Microsoft Excel.

## 3. Results

**Figure 1:**
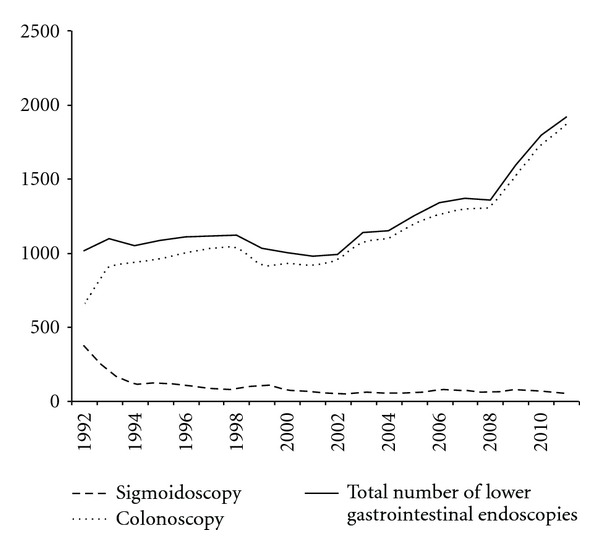
The yearly number of sigmoidoscopies and colonoscopies.

In the period of 20 years a total of 24431 endoscopies of the lower digestive tract were done. Per year this was mean 1222, range 977–1912. The yearly number of sigmoidoscopies had a mean 96 and range of 42–370. The mean number of colonoscopies was 1126, range of 643–1912. The yearly number of endoscopies is seen in Figure 1. The number of colonoscopies significantly increased, while the number of sigmoidoscopies decreased accordingly.

**Figure 2:**
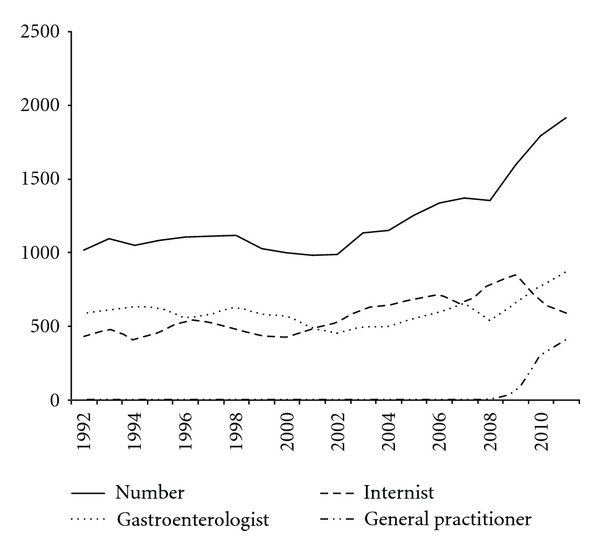
The number of endoscopies done on request of different specialists in the consecutive years.

The partnership Internal Medicine/Gastroenterology expanded in the course of the years. In 1992 it consisted of two gastroenterologists and four internists. Scince 2006 seven internists were partner. And in 2010 a third gastroenterologist and two new internists joined the partnership. In this context Figure 2 has to be interpreted. The number of colonoscopies on request of an internist or gastroenterologist showed a slow but steady increase. Given the fact that the number of internists was higher, it can be concluded that gastroenterologists requested much more endoscopies in the course of the years. The number of endoscopies done on request of the cardiologist or surgeon was low, mean of 18 procedures per year, range of 0–67. In November 2009 an open access facility was opened for the general practitioner. As can be seen the number of colonoscopies done on request of the general practitioner showed a steep increase in two years.

From 1992 to September 2006 single shifts were done at the endoscopy department. In September 2006 double shifts were started. General availability of endoscopists is 40 weeks a year. Corrected for the number of endoscopists, in the twenty years a total of 7972 shifts were done. This results in a mean of three colonoscopies per shift.

**Figure 3:**
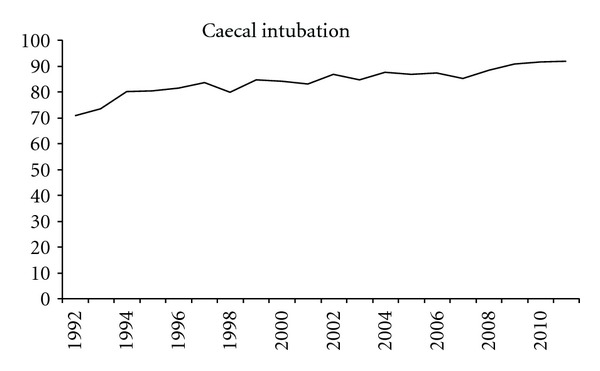
Successful caecal intubation in the consecutive years.

Figure 3 shows the percentage of successful caecal intubation in the twenty years. In 1992 caecal intubation occurred in 70% of the colonoscopies, and this number increased to 92% in 2011.

**Figure 4:**
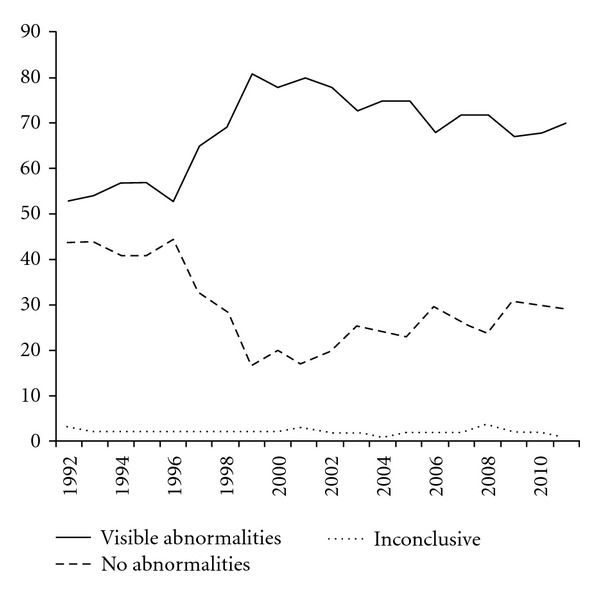
Macroscopic abnormalities and procedures with normal findings. An inconclusive endoscopy is defined as not possible due to inadequate colon cleansing.

Figure 4 shows the endoscopic findings in the consecutive years. In 1996 there was a steep increase in the percentage of procedures with abnormalities. Since 1999 the percentage of endoscopies showing abnormalities showed a slow decrease. The percentage of inconclusive procedures, mostly due to insufficient colon preparation, was low and stayed constant over the years.

**Figure 5:**
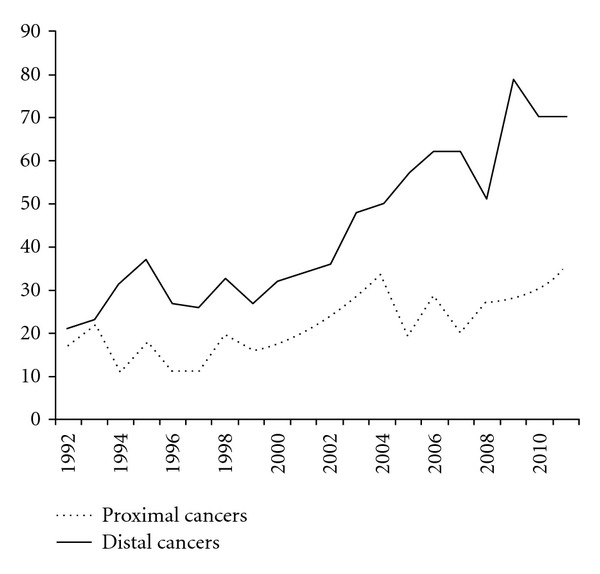
The number of proximal and distal cancers in the consecutive years.

**Figure 6:**
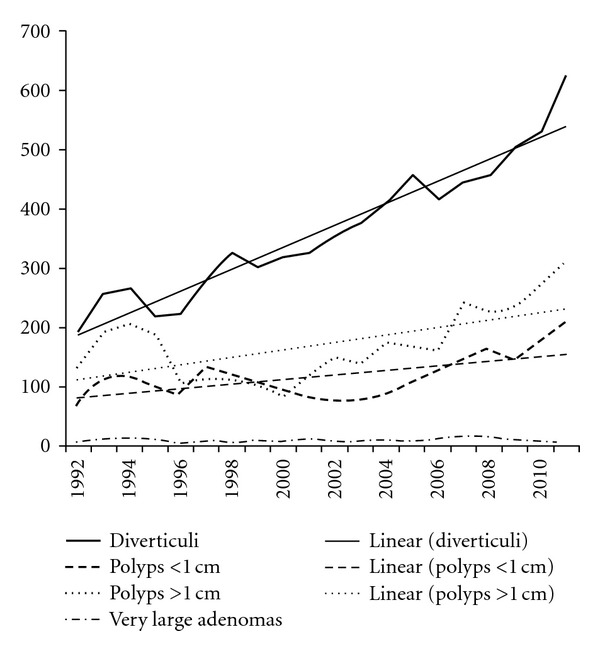
Polyps, large adenomas, and diverticuli with trend lines.

Figure 5 shows the amalgamation of proximal and distal (distal to the splenic flexure) cancers. The numbers of cancer increased in twenty years.  Figure 6 shows an increase in the number of polyps, smaller than 1 cm as well as larger than 1 cm. In addition, the number of large sessile adenomas stayed rather constant. The prevalence of diverticuli increased.

**Figure 7:**
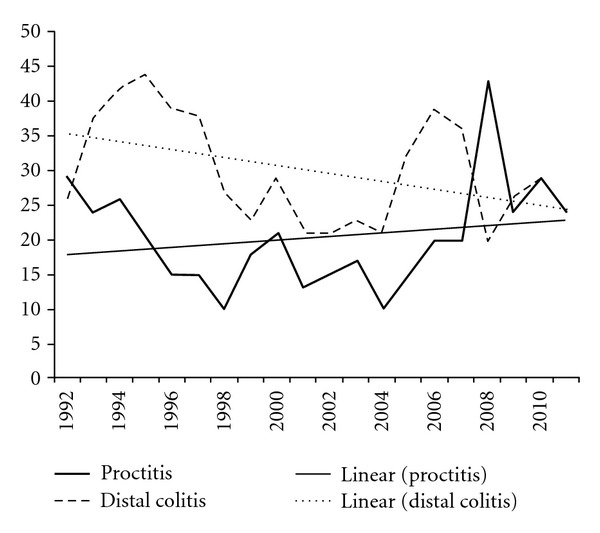
Proctitis and distal colitis in the consecutive years with trend lines.

**Figure 8:**
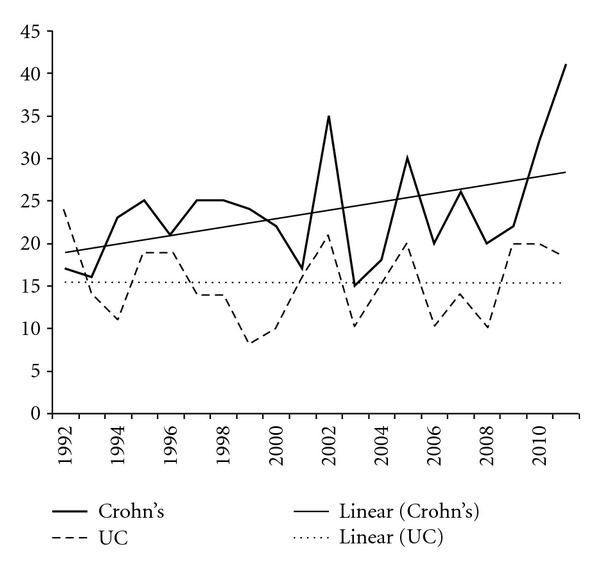
Crohn's disease and ulcerative colitis with trend lines.

Figures 7 and 8 show the numbers of procedures with signs of inflammatory bowel disease. The trend lines for Crohn's disease and proctitis show a slight increase, distal colitis showed a decrease, while the line for ulcerative colitis did not change.

## 4. Discussion

This study presents the results of endoscopy of the colon and rectum in a middle-sized Dutch hospital. The number of endoscopic procedures increased in the course of twenty years. The major reason for this increase is the expansion of endoscopists and the rise in number of weekly endoscopy shifts.

In the beginning of the nineties sigmoidoscopy was done in a large number of cases. Investigations of the colon were still done with barium enemas. Since 1994 the number of colonoscopies increased significantly, and the increment in endoscopic procedures is the result of these colonoscopies. Nowadays sigmoidoscopies are merely reserved for endoscopic diagnoses that require short-term followup, for instance inspection of the resection site of a large polyp, continuing resection of an adenoma or demarcation of the malignancy with ink.

It is beyond discussion that inspection of the entire colon is of the utmost importance. The percentage of successful caecal intubations increased in the course of the years. The reasons for this are obvious: first the experience of the endoscopists, but also the introduction of newer endoscopes which are much longer compared with the old fibre-optic endoscopes and the EVIS 100 series. Of course it is not always possible to reach the caecum, for instance due to obstructing tumours. But on the other hand the endoscopists also can decide to stop the procedure before reaching the caecum because they are satisfied with the findings seen in the distal colon, for instance inspection of therapeutic success in case of distal colitis or proctitis. An earlier study from our group reported on the clinical reason for unsuccessful colonoscopy [2].

The numbers of endoscopies requested by the gastroenterologist or internists showed little fluctuation in 20 years. But because of the expansion of the staff with more internists, it can be concluded that gastroenterologists requested relatively more procedures. Every internist has a specific interest like oncology, hematology, endocrinology, or nephrology, but every specialist also practices general internal medicine. The introduction of the open-access facility for general practitioners is a great success. The number of requested procedures showed a steep increase. Rainis et al. studied the results of open access colonoscopy endoscopy without prior gastrointestinal consultation. In a period of 33 months 10866 colonoscopies were performed. 3533 pathologic findings were found, in 2978 colonoscopies. Rate of colonoscopies “generally indicated” according to American Society for Gastrointestinal Endoscopy guidelines was 78% with a rate of colonoscopies “generally not indicated” of 22%. It was concluded that open access colonoscopy is a reliable and safe method for screening average risk population [3]. The local findings with open access colonoscopy are in accordance with these data [4]. 

In the beginning of the nineties the number of procedures revealing no abnormalities was around 45%. The number of endoscopies with macroscopic abnormalities showed a significant increase. The most possible reason for this is the increase in colonoscopies and the decreasing numbers of sigmoidoscopies. In the final 5 years the number of endoscopies with no abnormalities slightly increases. A possible explanation can be that many more patients are in followup after prior removed adenomas. Many of these patients have a colon with normal anatomy. This observation was also done in a study by Laiyemo et al. They examined use and yield of surveillance colonoscopy among participants in the Polyp Prevention Trial. Seven hundred seventy-four subjects (59.7%) had a repeat colonoscopy. Surveillance colonoscopy was overused for low-risk subjects and underused for high-risk subjects. Advanced adenoma yield corresponded with the adenoma risk category [5]. 

Obviously, all patients in the present study had a clinical reason for endoscopy. Unfortunately the reason was not noted in the data set. Lasson et al. assessed the outcome of colonoscopy in patients with various gastrointestinal symptoms. The diagnostic yield of colonoscopy was high in patients with bleeding symptoms or diarrhoea, while the prevalence of significant findings was equal to a screening population in patients with other symptoms [6]. Goners et al. did an observational study in order to examine the relationship between appropriateness criteria and diagnostic yield of colonoscopy. Significant diagnoses included cancer, adenomatous polyps, angiodysplasia, and new diagnoses of inflammatory bowel disease. Twenty four percent of 5213 patients had significant diagnoses made, including 4% cancers and 14% adenomatous polyps. Among patients who had a significant diagnosis, 53% had an appropriate indication, 25% had an uncertain indication, and 22% had an inappropriate indication [7]. 

The number of colorectal cancers increased in the study period. This is in accordance with data from the local as well as the national cancer registries in The Netherlands [8, 9]. The same was true for diverticulosis of the colon. The main reason for this increase, obviously, is aging of the population.

Also the numbers of patients with polyps, large as well as small, show a definite increase. This observation has major implications for future practice. It can be expected that asymptomatic people with polyps will have a positive FIT test for occult bleeding. Since all patients in the present study had a clinical reason for the endoscopy and screening is not yet done in The Netherlands, with the exception of some research studies, these screenies have to undergo colonoscopy. Niv et al. did a meta-analysis of the prospective cohorts using total colonoscopy for screening asymptomatic individuals. Ten studies of screening colonoscopy conducted in asymptomatic people, with a total of 68,324 participants, were included. Colorectal cancer was found in 0.78% of the participants. Advanced adenoma was found in 5% of the cases [10]. Data from The Netherlands are not much different [11]. These numbers will add up to the already diagnosed polyps. It can be expected that the work-load will increase. One can argue whether endoscopists working in normal daily practice will find the time for all these screening colonoscopies. 

The trend line for Crohn's disease shows an increase. This is in accordance with the rising prevalence of this disease reported in the literature [12]. Ulcerative colitis as well as distal colitis shows a slow decrease in the twenty years. A reason for this observation is not obvious.

It can be concluded that the diagnostic yield of colonoscopy is high. Especially the numbers of patients with colorectal cancer, proximal as well as distal, and the patients with polyps show a significant increase in the course of the years. It will be interesting to expand this dataset in the future. Maybe the result of all polyepectomies in the past will lead to a decrease in the numbers of diagnosed colorectal cancer in the future.
